# Cocaine-Induced Rhabdomyolysis With Acute Kidney Injury in the Absence of Serotonin Syndrome: A Case Report

**DOI:** 10.7759/cureus.101177

**Published:** 2026-01-09

**Authors:** Mojeed Adiat, Samraiz Nafees, Vikram Kumar, Mohamed Abdelfattah, Ali Javeed, Muhammad Mubeen Anwar, Khaled Zamari, Muhammad Haroon Riasat, Muhammad Tahir Chohan

**Affiliations:** 1 Internal Medicine, York and Scarborough Teaching Hospital NHS Foundation Trust, Scarborough, GBR; 2 Internal Medicine, Liaquat University of Medical and Health Sciences, Jamshoro, PAK; 3 General Practice, Gloucestershire Hospitals NHS Foundation Trust, Bath, GBR; 4 Diabetes and Endocrinology, York and Scarborough Teaching Hospital NHS Foundation Trust, Scarborough, GBR

**Keywords:** acute kidney injury, cocaine use, creatine kinase, myoglobin, rhabdomyolysis, serotonin syndrome

## Abstract

Rhabdomyolysis is a clinical condition resulting from skeletal muscle breakdown, leading to the release of intracellular components, such as creatine kinase, myoglobin, and electrolytes, into the circulation. This process can cause a range of complications, most notably acute kidney injury (AKI), due to myoglobin-induced renal toxicity. While trauma, seizures, and hyperthermia are well-known precipitants, stimulant drugs represent an underrecognized yet clinically significant cause.

This report describes a middle-aged patient who presented with marked rhabdomyolysis and acute kidney injury (AKI) following intranasal use of cocaine.* *Notably, the patient exhibited no features of serotonin syndrome despite concurrent use of an antidepressant medication. Laboratory investigations revealed markedly elevated creatine kinase levels, while prompt intravenous fluid resuscitation resulted in complete renal recovery without the need for dialysis.

This case highlights the diagnostic importance of considering stimulant-induced muscle injury in patients presenting with unexplained rhabdomyolysis and renal dysfunction, particularly when typical precipitating factors are absent. Early recognition and aggressive hydration remain key management strategies to prevent irreversible renal damage and improve outcomes.

## Introduction

Rhabdomyolysis is a clinical syndrome characterised by the breakdown of skeletal muscle fibres, leading to the release of intracellular constituents, such as creatine kinase (CK), myoglobin, and electrolytes, into the bloodstream. Creatine kinase serves as a key diagnostic marker, while myoglobin release can result in myoglobinuria and direct tubular injury, predisposing to acute kidney injury (AKI). The clinical consequences range from asymptomatic biochemical elevations to life-threatening complications such as acute renal failure, cardiac arrhythmias, and severe metabolic disturbances [1].

The condition has a broad spectrum of causes, including trauma, excessive physical exertion, seizures, heat stroke, drug misuse, and exposure to toxins. Among drug-related aetiologies, stimulant substances, such as cocaine, are well-recognised contributors. Cocaine exerts potent sympathomimetic effects that promote vasoconstriction, increase energy expenditure, and predispose muscle tissue to hypoxia and subsequent breakdown [2]. Despite this well-described mechanism, cocaine-induced rhabdomyolysis remains an underdiagnosed clinical entity, accounting for a small but significant proportion of drug-related AKI cases reported in the literature [3,4].

This report describes a rare and severe case of rhabdomyolysis with AKI following isolated intranasal cocaine use, occurring in the absence of more typical precipitating factors such as hyperthermia, seizures, or trauma. The patient was concurrently prescribed sertraline, which prompted consideration of serotonin syndrome at presentation; however, this was excluded based on the absence of characteristic neuromuscular and autonomic features. The case underscores the importance of maintaining a high index of suspicion for drug-induced muscle injury in patients presenting with unexplained renal dysfunction or markedly elevated CK levels. The report follows a structured description of the clinical presentation, investigations, management, and patient outcome.

## Case presentation

A 41-year-old man presented to the emergency department with a two-day history of generalised malaise, agitation, confusion, and progressively worsening drowsiness. He also noted the recent development of an erythematous rash across the nasal bridge and forehead, which he attributed to recent intranasal cocaine use. His medical history included obstructive sleep apnoea, managed with continuous positive airway pressure (CPAP) therapy, and depression, managed with sertraline 100 mg once daily. He denied recent trauma, seizures, excessive exertion, or alcohol intake.

On further questioning, the patient stated that he had been using street cocaine (“sugar”) intranasally on and off for recreational purposes over the preceding three months, with no other illicit drug use reported and no previously documented history of substance abuse.

Initial laboratory investigations are summarised in Table 1. Laboratory evaluation revealed marked abnormalities consistent with severe rhabdomyolysis and early acute kidney injury. Creatine kinase (CK), alanine aminotransferase (ALT), serum creatinine, white blood cell count, and C-reactive protein (CRP) were all increased. Multiple arterial blood gas (ABG) analyses were performed, mainly in view of his initially deranged renal function tests and elevated creatine kinase levels. Lactate levels were consistently within the normal range on all occasions. Blood and urine cultures showed no microbial growth, and viral screening was negative. Neuroimaging with brain CT and chest X-ray did not reveal any acute abnormalities. Cerebrospinal fluid (CSF) analysis obtained via lumbar puncture was unremarkable.

**Table 1:** Admission investigations revealed markedly elevated CK levels consistent with rhabdomyolysis, accompanied by rising creatinine and inflammatory markers

Parameter	Result	Reference Range
Creatine Kinase (CK)	18,753 U/L	40–320 U/L
Alanine Aminotransferase (ALT)	240 U/L	0–45 U/L
Creatinine	152 µmol/L	59–104 µmol/L
White Cell Count	11.03 ×10⁹/L	4–11 ×10⁹/L
C-Reactive Protein (CRP)	155 mg/L	<5 mg/L

The patient was admitted for close observation and management of rhabdomyolysis and potential renal impairment. Aggressive intravenous hydration was initiated immediately, with careful monitoring of urine output, renal function, and serum electrolytes. Over the following 72 hours, his mental status improved progressively. Serial laboratory monitoring demonstrated a steady decline in CK, creatinine, and inflammatory markers (Table 2). Renal replacement therapy was not required. The patient was discharged in stable condition and was referred to addiction services for ongoing support and counselling.

**Table 2:** Serial laboratory investigations demonstrated progressive biochemical improvement with declining CK, creatinine, and inflammatory markers following aggressive intravenous hydration and supportive management

Parameter	Reference Range	21-08-2025 00:24	21-08-2025 20:03	22-08-2025 08:17	23-08-2025 08:20
Creatine Kinase (CK) U/L	40–320	18,753	10,005	6,961	2,109
Alanine Aminotransferase (ALT) U/L	0–45	40	269	284	263
Creatinine (µmol/L)	59–104	152	100	83	82
C-Reactive Protein (CRP) (mg/L)	<5	155	126	74	38

## Discussion

Cocaine-associated rhabdomyolysis represents a complex clinical entity with multifactorial pathophysiology. Three major mechanisms are frequently described: intense vasoconstriction leading to muscle ischemia, direct myotoxicity from cocaine and its metabolites, and catecholamine-induced hyperadrenergic states that increase muscle activity and energy depletion. These mechanisms often interact synergistically to produce widespread muscle injury. Less common yet significant contributors, such as cocaine-induced vasculitis, focal infarction, and microangiopathic changes, have also been reported and may explain the variability in disease severity among individuals exposed to similar doses [5].

Although cocaine-related muscle injury is often described as mild or moderate in toxicology studies, severe rhabdomyolysis with strikingly elevated creatine kinase (CK) levels and acute kidney injury (AKI) has been documented in isolated case reports and small series [6]. Most of these severe presentations occur in the context of additional precipitating factors, including prolonged immobilisation, generalised seizures, trauma, hyperthermia, or co-ingestion of other sympathomimetic agents. The present case is notable for its severity following isolated intranasal cocaine exposure, without these typical external triggers. It reinforces that non-injectable routes of administration can still yield substantial systemic absorption, intense adrenergic effects, and profound myotoxicity [7].

The possible interaction between serotonergic antidepressants and cocaine remains an area of clinical interest. Co-administration may theoretically amplify the risk of serotonin syndrome through synergistic increases in serotonergic transmission [8]. However, the presence of a selective serotonin reuptake inhibitor (SSRI) alone is neither sufficient nor required to induce serotonin toxicity. Diagnosis remains clinical and relies on features such as clonus, hyperreflexia, hyperthermia, and muscle rigidity. In this case, although the patient was taking sertraline, the absence of these clinical signs excluded serotonin syndrome, supporting the understanding that SSRI use may increase theoretical risk but does not invariably result in serotonin-mediated toxicity [9].

Early recognition of cocaine-induced rhabdomyolysis is critical, as initial symptoms may be subtle or nonspecific. Management centres on prompt, aggressive intravenous hydration to maintain renal perfusion, achieve high urine output, and prevent myoglobin-induced nephropathy. Electrolyte monitoring, particularly for hyperkalaemia, metabolic acidosis, and evolving renal dysfunction, is essential. Most patients recover with timely fluid therapy, whereas delayed management increases the risk of dialysis-requiring AKI.

Clinical outcomes vary widely and correlate with the degree of biochemical derangement, delay in treatment initiation, baseline health status, and concurrent insults. Many patients achieve full recovery with supportive measures alone, but others experience prolonged renal impairment or require temporary renal replacement therapy. Importantly, intranasal cocaine use should not be perceived as a benign route, as systemic absorption is sufficient to produce severe vasoconstriction, adrenergic stress, and direct muscular damage.

This case highlights several key learning points. Clinicians should maintain a high index of suspicion for stimulant-related muscle injury when assessing patients with unexplained rhabdomyolysis or renal dysfunction, even in the absence of classic triggers such as trauma or seizures. Toxicology screening should be performed early, and fluid resuscitation initiated promptly. In atypical or refractory cases, alternative mechanisms, such as vasculitis or thrombotic microangiopathy, should be considered [10]. Increased awareness and early intervention remain essential to prevent irreversible renal damage and improve outcomes in patients with cocaine-induced rhabdomyolysis.

## Conclusions

Cocaine-induced rhabdomyolysis, especially when complicated by acute kidney injury, can develop even in the absence of commonly recognised precipitating factors such as serotonin syndrome, seizures, trauma, or hyperthermia. The potential for severe systemic toxicity exists, irrespective of the route of cocaine administration or the presence of other classical risk factors. This case highlights the crucial need for clinicians to consider cocaine toxicity in acutely unwell patients who present with neuropsychiatric symptoms, unexplained elevations in creatine kinase, or acute kidney dysfunction.

Prompt recognition, early toxicology screening, and initiation of aggressive intravenous fluid resuscitation, combined with close monitoring of renal and metabolic parameters, remain the cornerstone of effective management. Early and decisive intervention can significantly reduce the risk of renal failure and prevent long-term sequelae. Sustained clinical vigilance and a proactive approach to screening and fluid resuscitation are essential to improving outcomes in suspected cases of drug-induced rhabdomyolysis.
